# SysWiz database and software tool providing an insight into mechanism of action of molecules associated with ciliopathies

**DOI:** 10.1186/2046-2530-1-S1-P12

**Published:** 2012-11-16

**Authors:** G Apic

**Affiliations:** 1 Cambridge Cell Networks Ltd, UK

## 

SysWiz Database, the online database from the Cambridge Cell Networks Ltd. (CCNet) tailored for the purpose of FP7 Syscilia project with a systems biology approach to studying cilia, has integrated manually curated and interpreted information from public domain (scientific literature) related to ciliary functions and diseases - list of protein (gene) interactions as well as list of ciliopathies and associated genes – around 3000 of them, involved in different ciliary diseases, curated and interpreted from the literature (PubMed) by CCNet. Furthermore, some preliminary legacy data from different partners within the FP7 Syscilia project with systems biology approach to studying cilia, such as lists of ciliopathies (syndromes) with associated genes. SysWiz database/software tool provides you with information on what proteins/genes/chemicals are associated with specific ciliary diseases, with a number of supporting literature references beside each molecule and hyperlinks to abstracts on PubMed. SysWiz offers visualized relationships between the molecules, associated with specific ciliopathy, as well as related chemicals from assay of partner with supporting literature references by clicking on the connector lines and hyperlinks to abstracts on PubMed. By using the SysWiz integrated system, one can obtain an in-depth understanding of possible mechanism of action as well as to extrapolate data and observed effects from other species into humans.

